# The feasibility of measuring and monitoring social determinants of health and the relevance for policy and programme – a qualitative assessment of four countries

**DOI:** 10.3402/gha.v9.29002

**Published:** 2016-02-05

**Authors:** Erik Blas, John E. Ataguba, Tanvir M. Huda, Giang Kim Bao, Davide Rasella, Megan R. Gerecke

**Affiliations:** 1 International Public Health Consultant, Copenhagen, Denmark; 2 Health Economics Unit, School of Public Health & Family Medicine, University of Cape Town, Cape Town, South Africa; 3 School of Public Health, University of Sydney, Sydney, Australia; 4 Centre for Child and Adolescent Health, icddr,b, Dhaka, Bangladesh; 5 Institute of Preventive Medicine and Public Health, Hanoi Medical University, Hanoi, Vietnam; 6 Institute of Collective Health, Federal University of Bahia, Salvador, Brazil; 7 Social Determinants of Health, World Health Organization, Geneva, Switzerland

**Keywords:** sustainable development goals, universal health coverage, equity, human rights, gender, intersectoral action

## Abstract

**Background:**

Since the publication of the reports by the Commission on Social Determinants of Health (CSDH), many research papers have documented inequities, explaining causal pathways in order to inform policy and programmatic decision-making. At the international level, the sustainable development goals (SDGs) reflect an attempt to bring together these themes and the complexities involved in defining a comprehensive development framework. However, to date, much less has been done to address the monitoring challenges, that is, how data generation, analysis and use are to become routine tasks.

**Objective:**

To test proposed indicators of social determinants of health (SDH), gender, equity, and human rights with respect to their relevance in tracking progress in universal health coverage and population health (level and distribution).

**Design:**

In an attempt to explore these monitoring challenges, indicators covering a wide range of social determinants were tested in four country case studies (Bangladesh, Brazil, South Africa, and Vietnam) for their technical feasibility, reliability, and validity, and their communicability and usefulness to policy-makers. Twelve thematic domains with 20 core indicators covering different aspects of equity, human rights, gender, and SDH were tested through a review of data sources, descriptive analyses, key informant interviews, and focus group discussions. To test the communicability and usefulness of the domains, domain narratives that explained the causal pathways were presented to policy-makers, managers, the media, and civil society leaders.

**Results:**

For most countries, monitoring is possible, as some data were available for most of the core indicators. However, a qualitative assessment showed that technical feasibility, reliability, and validity varied across indicators and countries. Producing understandable and useful information proved challenging, and particularly so in translating indicator definitions and data into meaningful lay and managerial narratives, and effectively communicating links to health and ways in which the information could improve decision-making.

**Conclusions:**

This exercise revealed that for monitoring to produce reliable data collection, analysis, and discourse, it will need to be adapted to each national context and institutionalised into national systems. This will require that capacities and resources for this and subsequent communication of results are increased across countries for both national and international monitoring, including the successful implementation of the SDGs.

## Introduction

The Millennium Development Goals (MDGs), formulated at the end of the 20th century, focused on a select number of development indicators and helped to achieve remarkable reductions in, for example, the burden of malaria and tuberculosis. In the first decade of the 21st century, the health, scientific, and political community increasingly looked upon development more holistically (1). In 2005, the World Health Organization (WHO) Commission on Social Determinants of Health (CSDH) was established (2). Social determinants of health (SDH) influence the health of populations through different pathways. The circumstances in which people grow, live, work, and age have a direct impact on the level and distribution of health in populations (Pathway A). However, social determinants also impact access, provision, and ability to benefit from health services (Pathway B). Political, social, and economic forces in turn shape social determinants and health systems. Public health programmes, managers, and policy-makers must analyse, document, and reach beyond the health sector to inform other sectors in order to influence the political, social, and economic forces at the root of population health (3, 4).

The WHO's 12th General Programme of Work (2014–2019), entitled ‘Not merely the absence of disease’, paraphrased from the WHO constitution, includes “addressing the social, economic and environmental determinants of health as a means of reducing health inequities within and between countries” as one of its six leadership priorities. It further aims to have “gender, equity and human rights integrated into the Secretariat's and countries’ policies and programmes” (5). The sustainable development goals (SDGs), currently being endorsed as a successor of the MDGs, aim “to strive for a world that is just, equitable and inclusive … to promote sustained and inclusive economic growth, social development and environmental protection …” (6). While in the SDGs, health sits comfortably to “Ensure healthy lives and promote well-being for all at all ages” (Goal 3), the question arises as to what narrative exists for weaving together indicators for health determinants across other sectoral goals.

Reports and peer-reviewed literature describe many links between determinants and health equity. However, there is a need to better understand how indicators can be used to spur action and accountability across sectors (7). Early evidence suggests monitoring can be useful in this respect. For example, in reviewing the experience of monitoring in New Zealand, Pega et al. (8) noted that national-level social monitoring provides a valuable tool for raising awareness across government and civil society. Experiences in Norway (9) and Indonesia (10) also highlight the importance of making relevant sectoral agents responsible for reporting on social indicators. The call for greater emphasis on implementation science in relation to transdisciplinary and intersectoral action on social determinants and health equity is getting more articulate (11, 12). This paper answers this call, exploring the challenges posed by monitoring the social determinants.

A component of the WHO and Rockefeller-funded research-to-policy project (unpublished) focused on addressing how equity-oriented indicators of health determinants and barriers could be used to spur action among health and other sectoral policy-makers. The four case studies reported in this article tested a set of proposed indicators on equity, human rights, gender, and SDH with respect to: 1) technical feasibility, relevance, and validity; 2) reliability of data sources, and 3) policy and programmatic feasibility and relevance.

## Methodology

An initial framework for monitoring intersectoral factors influencing health with an equity-focus was developed through literature reviews and a consultative process (unpublished). It included 12 domains captured by 20 core and six non-core indicators. The domains and indicators fit the following criteria: 1) address both pathways ‘A’ and ‘B’; 2) capture barriers to health services viewed from the perspective of the population, and 3) include determinants and barriers that could only be redressed by intersectoral action, that is, the health sector working with another sector. Based on this framework, a common protocol was prepared for the four case studies to facilitate cross-case analysis. The protocol included the key structure of the framework, domain, and indicator definitions and standards by which the technical feasibility and reliability, validity and policy and programmatic feasibility and relevance of the indicators and domains are assessed.

The focus of this paper is a qualitative analysis of the case study results related to the 12 domains and 20 core indicators (Table 1). The indicators shown in Table 1 were suggested to have a particular strength in detecting either pathway A or pathway B, and were investigated for how well they described the different dimensions of inequality (stratifiers). The results of the quantitative analyses are reported in separate country-specific papers published in this supplement.

**Table 1:** Domains and core indicators tested

Domains		Core indicators	Relationship to (Pathway A – determinant) and health services (Pathway B – barrier)
1	Income and poverty	A: Proportion of households under **poverty line** (nationally defined) … with one or more persons over retirement age/with one or more children under 5	Determinant
		B: **Gini coefficient**/concentration index	Determinant
2	Knowledge and education	C: Percentage of **mothers** with children aged 0–15 completing secondary **education**	Determinant/barrier
3	Housing and infrastructure	D: Percentage of settlements/population (depending on source) without **four basic amenities** energy, safe water, sanitation, and waste collection	Determinants
4	Travel	E: **Travel time** to (a) outpatient and (b) inpatient facility	Barrier
5	Community and infrastructure	F: Investment in **health promotion** versus curative care	Barrier
		G: Percentage of communities or households covered by **integrated vector control** management	Barrier
		H: **Safety** in the neighbourhood	Barrier/determinant
6	Social protection and employment	I: Share of **informal employment** in total employment	Barrier/determinant
		J: Proportion of the poorest households who received external **economic support** in the last 3 months	Barrier/determinant
7	Early child development	K: Extent of **early child development** (0–8 years) policies in place in country (index)	Determinants
8	Gender norms	L: **Seeking permission** for utilising a specific health service	Barrier
		M: Percentage of females **less than 20 years** of age **at first childbirth**	Determinant
9	Participation	N: Involvement in **decision-making** in the health system	Barrier
		O: Percentage of people **confident about using the health system**	Barrier
10	Registration (institutional constraints)	P: **Birth registration** coverage index	Barrier/determinant
11	Accountability (institutional constraints)	Q: Percentage of individuals paying something **(payments) other than an official fee** to access health services	Barrier
		R: Existence of public administration **oversight/transparency authority** (or clear penalties for public officials who appropriate public funds)	Barrier
12	Discrimination	S: Perceived **experiences of discrimination**	Barrier
		T: **Discrimination in the law**: protection of equal rights across race/ethnicity, religion, gender, disability in a constitutional document or similar document at the level of state/provinces and nationally	Determinants

Bolded words are the short names for the indicators used in this article.

### Technical feasibility and data source reliability

Technical feasibility addresses the ease of acquiring, analysing, and interpreting the data. Indicators and stratifiers were rated by each study team as follows: *high* – existing data sources can already provide the information in the format required or will need only modest modifications to do so, and data are relatively easy to analyse and interpret; *medium* – there are already existing structures for information collection; however, considerable modifications to current protocols will be needed to fit the format required and/or data might be more difficult to analyse and interpret; *low* – there is no existing basis for data collection and it would require significant changes to acquire, for example, establishment of new structures, new surveys or change of legislation, and/or data are difficult to analyse and interpret.

Technical reliability refers to how the data sources can be relied on to provide accurate information at present and in the future. The following questions were addressed and summary narratives were written for each data source:

1. Are methods and measures scientifically sound and stable over time?
2. Are the number of missing data and errors acceptable?
3. Is the data collection and processing transparent with credible audit or peer-review in place?
4. Is it free of political interference (design, collection, analysis, and publication)?
5. Is the data collection regularly repeated and is the data collection cycle shorter than or comparable to the expected pace of change?
6. Are there upcoming regulations that could impede collection and use of the required data?
7. Is the financing for the data collection stable?
8. Is there local technical capacity present for continued data collection?

### Technical validity

In the context of these case studies, validity addresses how well an indicator captures the influence of social determinants on access and the level and distribution of health in populations. Validity can be grouped into three types, which address the following questions (13): *construct validity* – is the proposed indicator of a reasonable proxy measure for the domain in question?; *Internal validity* – does the phenomenon measured by the indicator have an effect via pathways A and/or B on access, and level and distribution of health?, and *External validity* – what is the scope for generalising findings to the country as a whole and to the whole domain, that is, beyond what is directly measured by the particular indicator? The assessment was based on ‘best judgment’ exercised independently by each member of the team using a high–medium–low rating. Differences in rating between team members were discussed and a common rating was agreed upon.

### Policy and programmatic feasibility and relevance

Policy and programmatic feasibility and relevance address the usefulness of the proposed indicators for key audiences, namely policy-makers, senior health sector managers, civil society leaders, and the media. A limited number of focus group discussions (FGDs) and key informant (KI) interviews were conducted (see Table 2). For each FGD, five to eight people at a similar level of seniority were selected from different sectors relevant to the domains. In Brazil, KI interviews were conducted with those who, for logistics reasons, could not participate in the FGDs. In Vietnam, a larger number of FGDs and KI interviews were conducted and a specific protocol was prepared to ensure greater representativeness of the key audiences. Feasibility addresses whether the messages from the indicators are communicable and comprehensible, while relevance addresses whether the messages are seen as useful, whether the audience feels something would be done differently if this sort of information was available, and finally whether it would be useful for intersectoral dialogue and action, and to inform the public debate.

**Table 2:** Overview of work done

	Bangladesh	Brazil	South Africa	Vietnam
Number of Core indicators assessed	20	20	20	20
Number of data sources assessed	9	7	8	8
Number of focus group discussions performed	1	2	None	4
Number of key informant – interviews performed	None	4	None	7

To test the indicators as levers for change, short narratives were constructed for each domain based on the data given by the relevant indicators. These narratives were presented in the FGDs and KI interviews. The narratives included the following elements: proximal determinant/barriers; how they affected groups across the social gradient; and if possible, how they affected health sector performance. Following the FGDs and KI interviews, the teams rated the domains’ feasibility and relevance. Again, the assessment was based on ‘best judgment’ done independently by each member of the team using a high–medium–low rating. Differences in rating between team members were discussed and a common rating was agreed upon.

Table 2 summarises the range of activities covered by each of the country case study teams.

### Cross-case analysis

All case study teams submitted reports, which described key results in the form of tables and textual analysis. The results from the four case studies were comparatively analysed through compiling information on the categorical ratings provided by the teams and calculating average scores, setting high=3, medium=2, and low=1. Textual data responding to specific questions were analysed by drawing out common themes.

### Ethical approval

Ethical approval was obtained for the Bangladesh, Brazil, and Vietnam studies following relevant national requirements. The South Africa study did not seek ethical approval and only analysed secondary data that were already available.

## Results

This section first presents the technical feasibility of indicators and stratifiers, and the reliability of the underlying data sources. It then addresses the validity of the indicators. Finally, the findings with respect to policy and programmatic feasibility and relevance will be presented.

### Technical feasibility of measurement

#### Indicators

Table 3 shows the technical feasibility ratings of the indicators. Only four of the proposed core indicators are rated as having high feasibility across all four case countries. They are (B) ‘Gini coefficient’, (C) ‘Mothers education’, (D) ‘Four basic amenities’, and (I) ‘Informal employment’. All other indicators were rated as having medium or low technical feasibility by at least one country team. For six indicators, i.e. (E) ‘Travel time’, (R) ‘Oversight/transparency authority’, (T) ‘Discrimination in the law’, (K) ‘Early child development’, (L) ‘Seeking permission’, (N) ‘Decision making’, (O) ‘Confident using the health system’, (Q) ‘Payments other than official fee’, and (G) ‘Integrated vector control’, the average scores for technical feasibility are two or below. Reasons for the lower ratings include large-scale data not being systematically collected and data only available at aggregate level, infrequently or in formats that are not analysable. For the indicators (N) ‘Decision making’, (O) ‘Confident using the health system’, and (Q) ‘Payments other than official fee’, while some of this information is available from small studies, it would require considerable modifications for larger surveys to include these indicators.

**Table 3:** Summary findings on technical feasibility of indicators (high–medium–low and average score)

Indicator	Average score (maximum 3.0)	Bangladesh	Brazil	South Africa	Vietnam
B: Gini coefficient	3.0	High	High	High	High
C: Mothers education	3.0	High	High	High	High
D: Four basic amenities	3.0	High	High	High	High
I: Informal employment	3.0	High	High	High	High
A: Poverty line	2.8	Medium	High	High	High
J: Economic support	2.8	High	High	High	Medium
P: Birth registration	2.8	High	High	High	Medium
M: Less than 20 years at first childbirth	2.5	High	High	Low	High
F: Health promotion	2.3	Medium	Medium	Medium	High
H: Safety	2.3	Medium	Medium	High	Medium
S: Experiences of discrimination	2.3	Medium	High	Medium	Medium
E: Travel time	2.0	Medium	Medium	Medium	Medium
R: Oversight/transparency authority	2.0	Medium	High	Medium	Low
T: Discrimination in the law	2.0	Medium	High	Medium	Low
K: Early child development	1.8	Low	Low	Medium	High
L: Seeking permission	1.8	High	Low	Low	Medium
N: Decision–making	1.5	Low	Medium	Low	Medium
O: Confident using the health system	1.5	Low	Medium	Low	Medium
Q: Payments other than official fee	1.5	Medium	Low	Low	Medium
G: Integrated vector control	1.3	Low	Low	Low	Medium

Four indicators (A, J, M, and P) were rated as having high technical feasibility by all but one country team. (A) ‘Poverty line’ was rated as medium by the Bangladesh team because the national household and expenditure survey did not clearly document the methodology used. (J) ‘Economic support’ and (P) ‘Birth registration’ were given lower ratings by the Vietnam team because large-scale regular surveys did not include the information required. The South Africa team rated (M) ‘Less than 20 years at first childbirth’ as having low technical feasibility because the only reliable source of this data was dated 1998.

Finally, seven indicators (F, H, S, R, T, K, and L) stand out with only one study giving the rating of high. (F) ‘Health promotion’ and (K) ‘Early child development’ indicators were rated high only by Vietnam. The Vietnam National Health Account is done annually and separates preventive from curative services. However, for Vietnam as well as the other countries, it was not possible to analyse the data by different administrative zones. While in Vietnam, the Multiple Indicator Cluster Survey (MICS), which includes data on early child development, has been conducted five times, in the three other countries such information was difficult to extract due to heterogeneity of the data. (H) ‘Safety’ was rated as having *high* feasibility in South Africa and as having *medium* feasibility in other three country studies. However, in all studies it would be necessary to make modifications to existing surveys, for example, national health and living standard surveys. (L) ‘Seeking permission’ was rated high in the Bangladesh study although the data were available only for access to health services in general, and not for specific health services. In Vietnam, data were available in small-scale studies, while larger-scale surveys could be modified to collect such data (e.g. the Vietnam Living Standard and Annual Labour Force surveys). Neither Brazil nor South Africa had such available data, reportedly because this was not seen as a big cultural issue.

For indicators (R) ‘Oversight/transparency authority’, (S) ‘Experiences of discrimination’, and (T) ‘Discrimination in the law’, only the Brazil study rated the feasibility as high probably because specific authorities and laws already exist. The other three country studies either found that existing structures would need considerable modification or that there was, at the time, no basis for such data collection.

#### Social stratification

Table 4 presents the results for the technical feasibility of the proposed stratifiers. Four of the proposed common stratifiers (income/wealth, sex, age, education) were rated as high in all countries. Of the country-specific stratifiers, only ‘administrative political/geographic location’ achieved an overall score of three. For religion, race, ethnicity, and minority group, ratings varied across country teams. Information on religion was present in most household surveys in Bangladesh and Vietnam, while such information was only occasionally collected in Brazil and South Africa. Race was rated high for Brazil and South Africa where self-reported race information is often collected in household and health surveys, but not, for example, in routine service data collection. In Bangladesh and Vietnam, information on race were rated as having low feasibility. Ethnicity and minority group information were rated low in all countries except in Vietnam, where such information is present in most household surveys. It is of note that all four countries rated at least one of these last four indicators as having high feasibility.

**Table 4:** Summary findings on technical feasibility of stratifiers (high–medium–low and average score)

Stratifiers	Average score (maximum 3.0)	Bangladesh	Brazil	South Africa	Vietnam
Common stratifiers					
Income/wealth	3.0	High	High	High	High
Sex	3.0	High	High	High	High
Age	3.0	High	High	High	High
Education	3.0	High	High	High	High
Urban/rural	2.8	High	Medium	High	High
Country specific stratifiers					
Administrative/political areas – geographic location	3.0	High	High	High	High
Religion	2.3	High	Medium	Low	High
Race	2.0	Low	High	High	Low
Ethnicity	1.5	Low	Low	Low	High
Minority group	1.5	Low	Low	Low	High

### Reliability of data sources

The data sources were assessed according to the eight criteria defined in the Methodology section and the results are presented in Table 5.

**Table 5:** Summary findings of reliability of the top five data sources for indicators – listed according to the number of indicators covered

Bangladesh		Brazil	South Africa	Vietnam
Bangladesh Demographic and Health Survey		National Household Sample Survey (PNAD)	The National Income Dynamics Study (NIDS)	Vietnam Living Standard Survey (VLSS)
	Indicators: C, D, E, L, M Assessment: Conducted by National Institute of Population Research and Training (NIPORT) in collaboration with MEASURE DHS Program by ICF International, USA. It is a highly reliable data source. The survey is conducted every 4–5 years	Indicators: A, B, C, D, (E)*, I, J, M, Assessment: Highly reliable: institutional source of information from the Brazilian Institute of Geography and Statistics (IBGE), robust data collection, reduced missing, transparent processes with credible audit, free of political interference, repeated on a yearly basis, micro-data publicly available, stable financing, high technical capacity for data collection*(E) The health supplement of the PNAD, to which H, N, and Q could also be added in the future	Indicator: C, H, J, P Assessment: Is a highly reliable data source for monitoring income and expenditure dynamics in South Africa. It is a panel dataset collected through the University of Cape Town. There is no political interference with data collection and dissemination. The panel is repeated every 2 years	Indicators: A, B, C, D, E, G, H, I, J, L, M, N, O Assessment: Is conducted by the General Statistics Office of Vietnam (GSO). This is national representative and reliable.it is collected every 2 years. Data collection and processing are transparent
Household income and expenditure survey		National Demographic Census	The General Household Surveys (GHS)	National census
	Indicators: A, B, L Assessment: Conducted by Bangladesh Bureau of Statistics (BBS), the national statistical organisation of Bangladesh. This is a reliable data source. Data collection is transparent	Indicators: A, B, C, D, I, J, M Assessment: Highly reliable institutional source of information from the Brazilian Institute of Geography and Statistics (IBGE) repeated every 10 years. Same assessment as above	Indicator: D, E, O Assessment: Conducted by Statistics South Africa (Stats. SA). It is a highly reliable annual data source. Data collection is transparent	Indicators: C, D, P Assessment: Data are collected by GSO every 10 years. Data collection is reliable
Bangladesh Health Facility Survey		National Health Survey (NHS)	The Income and Expenditure Survey (IES)	Multiple Indicator Cluster Survey (MICS)
	Indicators: H,S Assessment: Was implemented by the University of South Carolina through subcontract to Tulane University. The survey instruments have been developed based on the Balanced Score Card (BSC) framework as well as the WHO building blocks for health systems. This is a reliable data source	Indicators: E, H, N, Q, S Assessment: Highly reliable, institutional source of information from the Brazilian Institute of Geography and Statistics (IBGE) in collaboration with the Ministry of Health. It will substitute the HS of the PNAD. Same assessment as above	Indicator: A, B Assessment: Is a highly reliable 5-yearly data source. Data collection is by ‘Stats. SA’. Data collection is transparent. Data are used *inter alia* to compute national poverty levels, inequality levels, and for updating the consumer price index	Indicators: L, M, P Assessment: Is conducted by GSO and there have been 5 MICS surveys since 1995. International technical protocol has been applied. Data are reliable and data collection is transparent
The Labour Force Survey		National Program for Access and Quality Improvement in Primary Care (PMAQ)	The National Victim of Crime Survey	Vietnam Labour Force and Employment Survey (LBS)
	Indicator: I Assessment: The Bangladesh Bureau of Statistics conduct this survey every 4–5 years. The last survey was carried out in 2010. This is regarded as a reliable source of data regarding employment status of population aged 15 years and older	Indicators: E, N Assessment: Highly reliable, institutional source of information from the Ministry of Health. Robust data collection, reduced missing, transparent processes with credible audit, free of political interference, micro-data publicly available, repeated every 2 years, stable financing, high technical capacity for data collection	Indicator: H Assessment: Is a reliable data source conducted by the national statistical authority. It is useful for generating crime statistics in the country. It is collected roughly every 5 years	Indicator: I Assessment: Is conducted by GSO. Data collection is transparent, repeated every year, and reliable
The Urban Health Survey			The Quarterly Labour Force Survey (QLFS)	
	Indicator: J Assessment: Conducted by National Institute of Population Research and Training (NIPORT) in collaboration with MEASURE Evaluation, USA		Indicator: I Assessment: Is a technically reliable data source conducted by ‘Stats. SA’. It is used to generate labour market statistics in the country. Data collection and processing is transparent and very reliable	
Indicators from other sources deemed reliable: F and P				
Indicators with sources of uncertain reliability: G, K, N, O, Q, R, and T		Indicators with sources of uncertain reliability: F, G, K, L, O, P, R, and T	Indicators with sources of uncertain reliability: F, G, K, L, M, N, Q, R, S, and T	Indicators with sources of uncertain reliability: F, H, K, Q, R, S, and T

All countries had data sources for half or more of the proposed indicators that are deemed reliable by the study teams. For some indicators, the data were even available from multiple sources within the same country (Table 5). In Brazil and Vietnam, the trend has been to concentrate most data collection in large national surveys undertaken by the Brazilian Institute of Geography and Statistics and General Statistics Office of Vietnam, respectively. The trend in South Africa has been to have fewer indicators from the same survey. However, only two institutions produce the majority of indicator data: the University of Cape Town and Statistics South Africa (Stats. SA). Bangladesh was the only country in the four that collaborated with international partners for most of the indicator data.

A total of 13 of the 20 proposed indicators are deemed by one or more of the study teams to have data sources of uncertain reliability according to the assessment criteria. Three of these indicators: (K) ‘Early child development’, (R) ‘Oversight/transparency authority’, and (T) ‘Discrimination in the law’ were deemed by all four study teams as having no reliable source of data. A further three – (F) ‘Health promotion’, (G) ‘Integrated vector control’, and (Q) ‘Other than official fee’, were deemed by three of the teams as having data sources of uncertain reliability.

### Technical validity of measurement

Eleven out of the 20 indicators were given an overall average score of 2.5 or more for *construct validity* (Table 6). However, only (C) ‘Mothers education’, (D) ‘Four basic amenities’, and (J) ‘Economic support’ were consistently rated high by all teams as being a reasonable proxy measure for their respective domains. Another six indicators: (A) ‘Poverty line’, (B) ‘Gini coefficient’, (I) ‘Informal employment’, (M) ‘Less than 20 years at first child birth’, (P) ‘Birth registration’, and (S) ‘Experiences of discrimination’ were rated high by three and medium by one of the teams. For four of the indicators: (E) ‘Travel time’, (K) ‘Early child development’, (Q) ‘Payments other than official fee’, and (T) ‘Discrimination in the law’ views were divided.

**Table 6:** Summary table of validity assessment (high–medium–low and average score)

		Average score (maximum 3.0)				Bangladesh			Brazil			South Africa			Vietnam		
						
Domain	Indicator	Total	Construct	Internal	External	Construct	Internal	External	Construct	Internal	External	Construct	Internal	External	Construct	Internal	External
2	C: Mothers education	2.9	3.0	3.0	2.8	H	H	H	H	H	H	H	H	M	H	H	H
6	J: Economic support	2.9	3.0	3.0	2.8	H	H	H	H	H	H	H	H	M	H	H	H
12	S: Experiences of discrimination	2.8	2.8	3.0	2.8	H	H	H	M	H	H	H	H	M	H	H	H
1	A: Poverty line	2.8	2.8	3.0	2.5	H	H	H	H	H	M	M	H	M	H	H	H
3	D: Four basic amenities	2.8	3.0	2.8	2.5	H	H	H	H	H	H	H	M	M	H	H	M
10	P: Birth registration	2.8	2.8	2.8	2.8	H	H	H	H	H	H	M	M	M	H	H	H
4	E: Travel time	2.6	2.5	2.8	2.5	H	H	H	M	H	M	M	M	M	H	H	H
6	I: Informal employment	2.6	2.5	2.8	2.5	H	H	H	H	H	H	M	L	M	H	H	H
8	M: Less than 20 years at first childbirth	2.6	2.8	2.8	2.3	M	H	M	H	H	H	H	M	M	H	H	M
1	B: Gini coefficient	2.5	2.8	3.0	1.8	H	H	M	H	H	M	M	H	M	H	H	L
11	Q: Other than official fee	2.5	2.5	2.8	2.3	H	H	H	H	H	M	L	M	L	H	H	H
7	K Early child development	2.4	2.5	2.5	2.3	H	H	H	M	H	M	M	M	L	H	M	H
12	T: Discrimination in the law	2.4	2.5	2.8	2.0	H	H	H	H	H	M	M	M	M	M	H	L
11	R: Oversight/transparency authority	2.3	2.0	2.8	2.3	M	H	H	M	M	L	H	H	M	L	H	H
8	L: Seeking permission	2.3	2.3	2.5	2.0	H	H	H	M	M	L	L	M	L	H	H	H
5	F: Health promotion	2.1	2.0	2.3	2.0	M	H	H	M	M	L	L	L	L	H	H	H
5	H: Safety	2.1	2.0	2.3	2.0	M	M	M	M	M	M	M	H	M	M	M	M
9	N: Decision-making	2.1	2.0	2.3	2.0	M	H	M	H	M	M	M	M	L	L	M	H
5	G: Integrated vector control	2.0	2.0	2.3	1.8	M	H	H	M	M	L	L	M	L	H	M	M
9	O: Confident using the health system	2.0	2.0	2.3	1.8	M	H	M	H	M	M	M	M	L	L	M	M

Seven indicators were consistently rated medium or low for construct validity. This includes all indicators proposed for the domain of ‘Community and infrastructure’, that is, (F) ‘Health promotion’, (G) ‘Integrated vector control’ and (H) ‘Safety’. The South Africa study team questioned whether there is an optimal level of investment in health promotion versus curative care highlights that integrated vector control is only relevant in high transmission areas and finds the neighbourhood safety indicator overly focussed on crime rate rather than transport and built environment, which would have been a more appropriate proxy of the domain. The teams judged the two indicators (N) ‘Decision making’ and (O) ‘Confident using the health system’ proposed for domain (9) ‘Participation’, as not having a satisfactory level of construct validity. For example, the Vietnam team found that on their own, both indicators were too vague and opinion-based to provide robust proxies for the participation domain.

The indicators (L) ‘Seeking permission’ and (R) ‘Oversight/transparency authority’ were also assessed as having medium or low construct validity. For example, the South Africa team questioned the validity of seeking permission, given the large proportion of female-headed households and the stronger trend of health-seeking among females. In this case, ‘Health-seeking’ would not be a meaningful proxy for ‘Gender norms’. The teams from Bangladesh, Brazil, and Vietnam all highlighted that without information on effectiveness, the mere presence of an oversight/transparency authority does not say much about the ‘Accountability’ domain.

As shown in Table 6, 12 of the 20 proposed core indicators were rated high by at least three of the study teams with respect to *internal validity*. Two indicators (I) ‘Informal employment’ and (K) ‘Early child development’ were rated by two teams as having medium validity and one, (L) ‘Seeking permission’, was rated by one team as having low internal validity. However, five indicators in two domains were rated less favourably.

In the domain of (5) ‘Community and infrastructure’, most teams rated all indicators medium or low. One reason for the low rating was the different lag periods between promotional interventions and health outcomes. Another was that health promotion in the context of ‘Community and infrastructure’ would be undertaken mostly by disease-specific programmes and targeted to specific population groups and thus only applicable to subsets of the population, making it difficult to attribute changes in this indicator with the general level and distribution of health. Further, while the effectiveness of integrated vector control was demonstrated, it was mostly focused on endemic or epidemic areas, and thus the contribution to the overall burden of disease and overall health in many countries might be small (South Africa). Likewise, for Bangladesh, the team's view was that safe neighbourhoods did not have a strong relationship with access to health care in the country (pathway B) but that safety was relevant as a determinant (pathway A).

For domain (9) ‘Participation’, three teams rated both indicators as having medium internal validity. For example, while agreeing with the general principle of participation and engagement, the South Africa team was not convinced that the indicator ‘Decision making’ (N) had any meaningful relationship with access to healthcare services. The Brazil team was of the view that confidence in the health system, in general (O), is difficult to measure and define.

Generally, the teams rated the indicators less favourably with respect to *external validity* compared to the other two validity measures. No indicators were rated high by all four teams and only five are rated as high by three of the teams. For five domains: (5) ‘Community and infrastructure’, (7) ‘Early child development’, (8) ‘Gender norms’, (9) ‘Participation’ and (11) ‘Accountability’ all indicators received medium or low ratings.

Notably indicator (B) ‘Gini coefficient’ was poorly rated, scoring only 1.8 with respect to external validity. The teams explained that it only describes differences between groups and generally is not widely available at sub-national levels. Indicator (F) ‘health promotion’ in Domain 5 is difficult to generalise as it is broad, ill-defined, and variable across programmes, services, and administrative areas as well as across countries (Brazil and South Africa). Apart from the challenge of obtaining reliable information on (G) ‘Integrated vector control’ in particular in decentralised countries, the variance in epidemiological situation and heterogeneity of implementation limits the generalisability (Brazil and Vietnam). The last indicator for Domain 5, that is, (H) ‘Safety’, is rated medium by all four countries. The main reason given was that safety was only one part of the picture, usually self-reported and often confounded with contextual, cultural, and social perceptions about risk.

The medium and low ratings of the (K) ‘Early child development’ indicator by Brazil and South Africa were explained by the division between policies and implementation. The existence of policies is of little value unless they translate into implementation that is measured and monitored. In addition, their coverage was limited by eligibility rules, particularly in light of the high percentage of informal employment.

For domain (8) ‘Gender norms’, views were diverse. While Bangladesh and Vietnam rated the indicator (L) ‘Seeking permission’ high for all types of validity, South Africa and Brazil found it too vague and unable to capture many other issues affecting gender differences in health. The South Africa team suggested that the focus of indicator (M) ‘Less than 20 years at first child birth’ on female health issues meant that it was not generalisable to the whole gender norm domain. The Bangladesh and Vietnam teams were of the view that early childbirth could be a reflection of individual free will rather than one of constraining social gender norms.

The two indicators in domain (9) rated ‘Participation’ score 2.0 and 1.8. For (N) ‘Decision making’, the main explanation for the low rating was that people are unlikely to have objective views on what aspects of health system are key to health and that the participation of individuals has little impact on health system and policy decision-making (Bangladesh and South Africa). In Brazil, some users’ commissions and participation mechanisms already exist, however, their effectiveness has not been assessed. For (O) ‘Confident using the health system’, the teams saw confidence as being a part of participation or possibly a result of it, but they did not find that increasing or decreasing levels of confidence can be taken as a general proxy for changes in levels of participation.

Finally, for domain (11) ‘Accountability’, the teams were divided; Bangladesh and Vietnam rated the external validity of the two accountability indicators as high, while Brazil and South Africa rated them as medium and low. The South Africa team felt that the indicator (Q) ‘Other than official fee’ might provide an early warning of problems with accountability but could not be generalised to other, possibly more critical, accountability issues that are possibly more critical. Self-reported data may be culturally determined and unlikely to be representative. This view was supported by the Brazil team members who explained that corruption mostly occurs at higher levels, while the use of personal relationships to facilitate access is more widespread. Both teams agreed that while an ‘Oversight/transparency authority’ might be necessary for accountability to exist, its presence is not a sufficient guarantee for accountability, as there could be issues with effectiveness and competence.

### Policy and programmatic feasibility

Table 7 summarises the assessments of domain feasibility in Bangladesh, Brazil, and Vietnam based on the FGD and KI interviews.

**Table 7:** Summary findings on policy and programmatic feasibility (high–medium–low and average score)

Domain	Average score (maximum 3.0)	Bangladesh	Brazil	Vietnam
2 Knowledge and education	3.0	High	High	High
3 Housing and infrastructure	3.0	High	High	High
4 Travel	3.0	High	High	High
8 Gender norms	3.0	High	High	High
5 Community and infrastructure	2.7	High	Medium	High.
6 Social protection and employment	2.7	High	Medium	High
7 Early child development	2.7	High	Medium	High
10 Registration (institutional constraints	2.7	High	High	Medium.
12 Discrimination	2.7	High	High	Low
1 Income and poverty	2.3	Medium	High	Medium
9 Participation	2.0	Data not available	Medium	Medium
11 Accountability (institutional constraints)	2.0	High	Medium	Low

*Note*: South Africa did not do this part.

All three countries rated four of the domains (2, 3, 4, and 8) as high, meaning that they found them to be easy to communicate. Five domains (5, 6, 7, 10, and 12) recived an average score of 2.7, while three (1, 9, and 11) scored 2.3 or less.

Ratings vary considerably across domains and countries. Generally, the rating of ‘medium’ reflects variability in how target audiences reacted; some found the domains understandable, while others did not. However, this is not the case for (9) ‘Participation’ in Vietnam. Here, all four target audiences had difficulties in understanding the meaning and the relationship between participation and health outcomes and coverage. In Brazil, this was only true of the media audience. For (1) ‘Income and poverty’, the main difficulty (and thus the justification for the medium rating) was the challenge of communicating the Gini coefficient to target audiences in a way that they could understand (Bangladesh and Vietnam).

The only domains to receive a low rating from a country were those of accountability and discrimination (11 and 12). This was by the Vietnam team. Despite the low rating, the team rates these domains as having medium usefulness for the same four audiences (see Table 8). Explaining this apparent contradiction is the fact that policy-makers and health sector managers emphasised difficulties with the validity of indicators, that is, they doubted whether these indicators reflected the true situation in the country. Civil society leaders and media informants emphasised low general public awareness of these matters and suggested that it would therefore be very demanding to communicate this information.

**Table 8:** Summary findings on policy and programmatic relevance (high–medium–low and average score)

Domain	Average score (maximum 3.0)	Bangladesh	Brazil	Vietnam
1 Income and poverty	3.0	High	High	High
2 Knowledge and education	3.0	High	High	High
3 Housing and infrastructure	3.0	High	High	High
4 Travel	3.0	High	High	High
8 Gender norms	3.0	High	High	High
6 Social protection and employment	2.3	High	Medium	Medium
9 Participation	2.3	Medium	High	Medium
10 Registration (institutional constraints)	2.3	Medium	High	Medium
11 Accountability (institutional constraints)	2.3	High	Medium	Medium
12 Discrimination	2.3	Medium	High	Medium
5 Community and infrastructure	2.0	Medium	Medium	Medium
7 Early child development	1.7	Medium	Low	Medium

*Note*: South Africa did not do this part.

### Policy and programmatic relevance

The policy and programmatic relevance broadly addresses the usefulness of the information, that is; are the messages useful to the target audiences; will they act differently with this sort of information available; will the information help to support intersectoral dialogue, and will it be useful in the public debate? Table 8 summarises the study teams’ assessment, based on the FGDs and KI interviews.

All three of the study teams rated five domains (1, 2, 3, 4, and 8) as being highly relevant. Two domains (5 and 7) were rated as medium or low by all teams. The rest had average scores of 2.3. The prime reason for a medium rating was that members of the target audiences felt that acting on the information would be outside their sphere of influence. However, the relevance for intersectoral dialogue was recognised in all cases. For example, for (6) ‘Community and infrastructure’ and (7) ‘Early child development’, Vietnamese health sector managers thought that health sector could only address ‘a small piece of this big cake’ and the media people found that in a low resource setting it would be hard to address the issues related to this domain. On the other hand, politicians/policy-makers and civil society leaders found that systematic monitoring of the domain was exactly what they needed for the intersectoral action and public debates. None of the target audiences in Vietnam questioned the feasibility of these two domains (Table 7).

## Discussion

Indicator monitoring is only useful if it provides relevant information for those in a position to use it for action. This audience must be able to understand the message correctly – either the value of the indicator directly or as embedded into a narrative (in this case, domain narratives). Valid indicators from across sectors must be available to provide a reasonably good measure of the social determinants’ influence on the level and distribution of health in the population (14). Given that most of the data underlying such indicators must come from large surveys, data collection will be a costly exercise and thus, in order to justify the expense, a high overall score across the case countries would be required on all accounts.

Overall, we found 11 of the 20 indicators scored 2.5 or more with respect to overall validity as judged by the case study teams. These covered 9 of the 12 domains, that is: (1) ‘Income and poverty’, (2) ‘Knowledge and education’, (3) ‘Housing and infrastructure’, (4) ‘Travel’, (6) ‘Social protection and employment’, (8) ‘Gender norms’, (10) ‘Registration’, (11) ‘Accountability’, and (12) ‘Discrimination’. This high rating reflects the fact that these 11 indicators are perceived as reasonable proxy measures for the domain in question, have a clear impact on the level or distribution of health outcomes or coverage and are reasonably generalisable (i.e. construct, internal, and external validity).

However, only eight of the 20 indicators scored 2.5 or more with respect to technical feasibility, that is: (A) ‘poverty line’, (B) ‘Gini coefficient’, (C) ‘mothers education’, (D) ‘four basic amenities’, (I) ‘informal employment’, (J) ‘economic support’, (M) ‘less than 20 years at first childbirth’, and (P) ‘birth registration’. Encouragingly, all countries rated the technical feasibility of four of the proposed common stratifiers (income/wealth, sex, age, education) as ‘High’. In the context of equity in Universal Health Coverage, a recent publication stressed the importance and feasibility of including complementary dimensions of social stratification, namely income, sex, and urban/rural residence (15). It should be noted that the stratifier of urban/rural residence scored only a ‘Medium’ rating in the Brazil case study.

A total of 13 of the 20 proposed indicators were deemed by one or more of the case study teams as currently having data sources of uncertain reliability. This suggests that the need for capacity building with respect to data, monitoring, and accountability as outlined in SDG 17.18 and 17.19 (6) is acute in the least developed countries, and that such tasks likely go far beyond these countries’ current capacities.

While this evaluation of validity, feasibility, and reliability is obviously important, more important still is whether monitoring these indicators can effect change. The very different viewpoints and understandings of policy-makers and senior managers from health and other sectors present challenges in moving from rhetoric to action (16–18).

When the information was presented to the target audiences (politicians/policy-makers, senior health sector managers, civil society leaders, and media) in the form of domain narratives, most domains were seen as communicable and comprehensible. In fact, only three domains did not receive a score of 2.5 or more (Table 7). Of the three, the poor rating of (1) ‘Income and poverty’ was mainly due to difficulty in making the target audience understand the Gini coefficient. This may not be a surprise, as it has long been known that there is no simple decomposition available for the coefficient that can be used in empirical work (19). For the domains of (9) ‘Participation’ and (11) ‘Accountability’, challenges are more general. Teams found it was difficult for the target audiences to understand both the message and why the domain would be important to population health. Further, the validity and measurability of the ‘Participation’ indicators were questioned by all four case study teams.

While the target audiences in general understood the domains and their indicators quite well, they did not believe that access to this kind of information would make much difference in their own decision-making. In fact, on this measure, 7 of the 12 domains scores were less than the 2.5 benchmark. However, the remaining five (1) ‘Income and poverty’, (2) ‘Knowledge and education’, (3) ‘Housing and infrastructure’, (4) ‘Travel’, and (8) ‘Gender norms’ all scored well. The key obstacle was that the audiences were of the view that policy action is outside of their spheres of influence. Despite this finding, they generally acknowledge the relevance of the domains for intersectoral and public purposes.

The lessons that might be drawn from this are threefold. First, the nine indicators falling below a 2.5 mark on overall validity should be redefined or replaced by others at least surpassing the benchmark for construct, internal, and external validity. Second, communicating with policy-makers takes a special set of skills that may be different from skills required for specialised data collection and analysis. Effective communication demands knowledge of the different target audiences, a well-developed skill-set and capacity for communicating. Third, the target audiences need to be educated on and provided with instruments that explain how to use the information. For example, Health-in-All Policies.^1^ Lessons from South Australia and Finland suggested that it is possible for researchers and policy-makers to develop a means of achieving a shared understanding across disciplines and sectors (20, 21).

For several of the 12 indicators that scored less than the 2.5 with respect to technical feasibility (Table 4), it is very likely that it will be challenging to establish internationally comparable measures. Further, the reliability of the sources underlying a considerable number of the indicators is deemed to be uncertain in the four countries. This, however, does not necessarily mean that measures comparable over time cannot be established. All four countries appear to have the technical and institutional capacity to undertake and take ownership of data collection and eventual modifications to questionnaires and other instruments (Table 6). Going forward, the nexus of capacity building could either be found within countries or with external sources. On the one hand, integrating data collection and funding into national institutions and budgets would have a positive effect on the reliability of national data sources. On the other hand, dependency on external funding, technical capacity, and protocols might help facilitate internationally comparable indicators. However, in some cases, even in the best of circumstances, it might be difficult to achieve full comparability. A case in point is the ‘Urban/rural’ stratifier that is used extensively internationally, including within the World Health Statistics. This stratifier is rated ‘medium’ technical feasible in the Brazil case study (Table 3) as the distinction between the two is deemed unclear. With the rapid urbanisation taking place across the world, this might be the case in an increasing number of countries. The use of stratifiers such as religion, race, ethnicity, and minority group for international monitoring of the SDGs, for example, might also be questionable as the four case studies clearly showed that these stratifiers have different meanings in different contexts and marked differences in their technical feasibility (Table 3).

While the domain of (8) ‘Gender norms’ had the highest possible score for both policy and programmatic feasibility and relevance (Tables 7 and 8), the two indicators of this domain, that is (M) ‘Less than 20 years at first childbirth’ and (N) ‘Decision-making’ were among the most divisive indicators across the case study teams. Such diverging views possibly reflect broader socio-cultural differences between the four countries and perhaps even gender biases within the respective case study teams. For comparative purposes, this domain would need to be aligned with internationally agreed-upon standards. For indicator (M), the age reference could, for example, be set at 18 years bringing it in line with the Convention on the Right of the Child which has been signed and/or ratified by most countries.^2^

Each of the 12 domains tested in the four case studies is included in at least one of the 17 SDGs proposed by the UN Open Working Group (6). The proposal further sets an ambitious target (17.18): “By 2020, enhance capacity-building support to developing countries, including for least developed countries and small island developing States, to increase significantly the availability of high-quality, timely and reliable data disaggregated by income, gender, age, race, ethnicity, migratory status, disability, geographic location and other characteristics relevant in national contexts”. It is not clear which stratifiers were proposed as global and which were national. However, it is clear that efforts and additional resources for building capacity for monitoring will be required in many countries around the world. This will also pose a challenge for health programmes and systems as presumably the sub-goals will need to be monitored using the proposed stratifiers, including those under SDG3 “Ensure healthy lives and promoting well-being for all at all ages”.

### Limitations

Although the findings of these case studies might not be generalisable to all countries, the four case study countries represent different levels of economic development and different ways of organising societies. They may therefore be seen as illustrative of the range of opportunities and challenges in monitoring the SDH and health coverage in real-world country settings.

## Conclusions

A country planning to begin monitoring social determinants of inequity in health would need to work in parallel on the following three tracks. The *first track* includes deciding the domains to be monitored and communicated through, for example, population health profiles and other materials containing indicators and narratives. Both policy and programmatic feasibility and relevance are rated high in all countries for the domains of (2) ‘Knowledge and education’, (3) ‘Housing and infrastructure’, (4) ‘Travel’ and (8) ‘Gender norms’. These would provide a good first wave of domains to include in monitoring. The next wave could include (6) ‘Social protection and employment’ and (10) ‘Registration’, both of which also score relatively well on feasibility and relevance for the target audiences. The *second track* includes improving the validity of domain indicators. Initial focus should be on indicators from the chosen domains. This analysis reveals room for improvement in the indicators currently proposed for the domains mentioned above in the first and second waves, as none of the proposed indicators received the maximum score on all three types of validity.

The *third track* includes identifying which stratifiers or dimensions of inequity are relevant and feasible in the particular country context. This might be done through review of small studies or surveys that might not fulfil all the reliability criteria. Once selected, these dimensions will need to be incorporated into representative and reliable national data sources. Of the tested dimensions of inequity, it is only income, sex, age, and education that show a potential for international use. The others, including urban versus rural, have different meanings in different national contexts. This track also calls for additional resources and international comparative research as a contribution towards building capacity in countries for a concerted monitoring of the implementation of the SDGs.

## Footnotes

1 www.who.int/social_determinants/hiap_statement_who_sa_final.pdf

## Footnotes

2 www.treaties.un.org/doc/publication/mtdsg/volume i/chapter iv/iv-11.en.pdf
